# Self-cleaning and antibiofouling enamel surface by slippery liquid-infused technique

**DOI:** 10.1038/srep25924

**Published:** 2016-05-16

**Authors:** JiaLi Yin, May Lei Mei, QuanLi Li, Rong Xia, ZhiHong Zhang, Chun Hung Chu

**Affiliations:** 1 College & Hospital of Stomatology, Anhui Medical University, Key Lab. of Oral Diseases Research of Anhui Province, Hefei, 230032, China; 2 Faculty of Dentistry, University of Hong Kong, Hong Kong, 999077; 3 Department of Stomatology, the Second Hospital affiliated to Anhui Medical University, Hefei, 230601, China; 4 Department of Stomatology, the Hospital of Anhui Province, Hefei, 230001, China

## Abstract

We aimed to create a slippery liquid-infused enamel surface with antibiofouling property to prevent dental biofilm/plaque formation. First, a micro/nanoporous enamel surface was obtained by 37% phosphoric acid etching. The surface was then functionalized by hydrophobic low-surface energy heptadecafluoro-1,1,2,2-tetra- hydrodecyltrichlorosilane. Subsequent infusion of fluorocarbon lubricants (Fluorinert FC-70) into the polyfluoroalkyl-silanized rough surface resulted in an enamel surface with slippery liquid-infused porous surface (SLIPS). The results of water contact angle measurement, diffuse-reflectance Fourier transform infrared spectroscopy, and atomic force microscope confirmed that the SLIPS was successfully constructed on the enamel surface. The antibiofouling property of the SLIPS was evaluated by the adsorption of salivary protein of mucin and *Streptococcus mutans in vitro*, as well as dental biofilm formation using a rabbit model *in vivo*. The results showed that the SLIPS on the enamel surface significantly inhibited mucin adhesion and *S. mutans* biofilm formation *in vitro*, and inhibited dental plaque formation *in vivo*.

The microbial biofilm is defined as a community of microbial cells attached to surfaces in natural and anthropogenic environments; such cells are embedded in a matrix of extracellular polymeric substance^1^. Mature biofilms are resistant to various antimicrobial treatments. Dental plaque is a typical biofilm that can accommodate a variety of flora^2^. Common dental diseases, such as dental caries and periodontal diseases, are caused by dental plaque. Furthermore, an association has been suggested between the oral microbiota and systemic diseases, such as diabetes, cardiovascular disease, atherosclerosis, and complications during pregnancy^3^,^4^.

Treatment of adherent biofilm is difficult and costly. In clinic dentistry, dental plaque is usually controlled by meticulous mechanical oral hygiene. However, most individuals can hardly maintain the necessary standards of plaque control strictly for a long time. Additional approaches are being developed that are less dependent on the dexterity of the patient, and which augment conventional oral hygiene methods and keep plaque at levels compatible with oral health. Consequently, a wide range of antimicrobial agents, such as chlorhexidine, triclosan, metal salts, enzymes, and plant extracts, have been formulated into oral care products to enhance their plaque control potential^5^,^6^,^7^,^8^. However, following the prolonged and persistent usage of biocidal chemotherapeutics, oral bacteria can potentially acquire resistance to these agents. Most importantly, such agents may be harmful to the normal, resident oral microflora, possibly resulting in new problems^9^. Therefore, the approach that prevents the initial attachment of bacteria is likely to be better than the antimicrobial approach that aims at killing bacteria already attached. The prevention of biofilm formation without using biocidal agents rather than treatment of biofilm using antimicrobials is highly desirable. Dental plaque formation begins with the bacteria recognizing the salivary pellicle, which is an acquired pellicle for salivary proteins adsorbed onto the dental surface^10^. Thus, to prevent dental biofilm formation, scientists should focus on inhibiting bacterial adhesion and salivary protein adsorption on the dental surface.

Common methods to construct the antibiofouling surface for medical devices and implants include functionalizing the surface chemical functional groups and then grafting poly (ethylene glycol) (PEG) or its analog, such as N-substituted glycine and polysarcosine^11^,^12^,^13^. However, none have yet proven ideal for long-term antibiofouling property. These antibiofouling surfaces are believed to be in solid forms. Permanent interactions between the solid surfaces and biological adhesives can eventually be established depending on the time scales of the adhesion processes, which then lead to stable attachment and biofouling^14^. Recently, a slippery liquid-infused porous surface (SLIPS) was introduced. The stabilized liquid interface possesses dynamic features down to the nanometer scale and may inhibit these permanent interactions, thereby significantly disrupting biological adhesion^15^,^16^,^17^. The antibiofouling effect of SLIPS is better than that of the PEGylated surface^18^. However, oral environment and oral bacterial flora are highly complex, and dental plaque formation exhibits distinctive behavior. To date, SLIPS on an enamel surface has not been reconstructed nor has its effect on inhibiting dental biofilm formation been evaluated. Thus, in the present study, we aimed to create a slippery liquid-infused enamel surface and evaluate its antibiofouling property.

## Results

### Characterizing the slippery liquid-infused enamel surface

**Figure 1:**
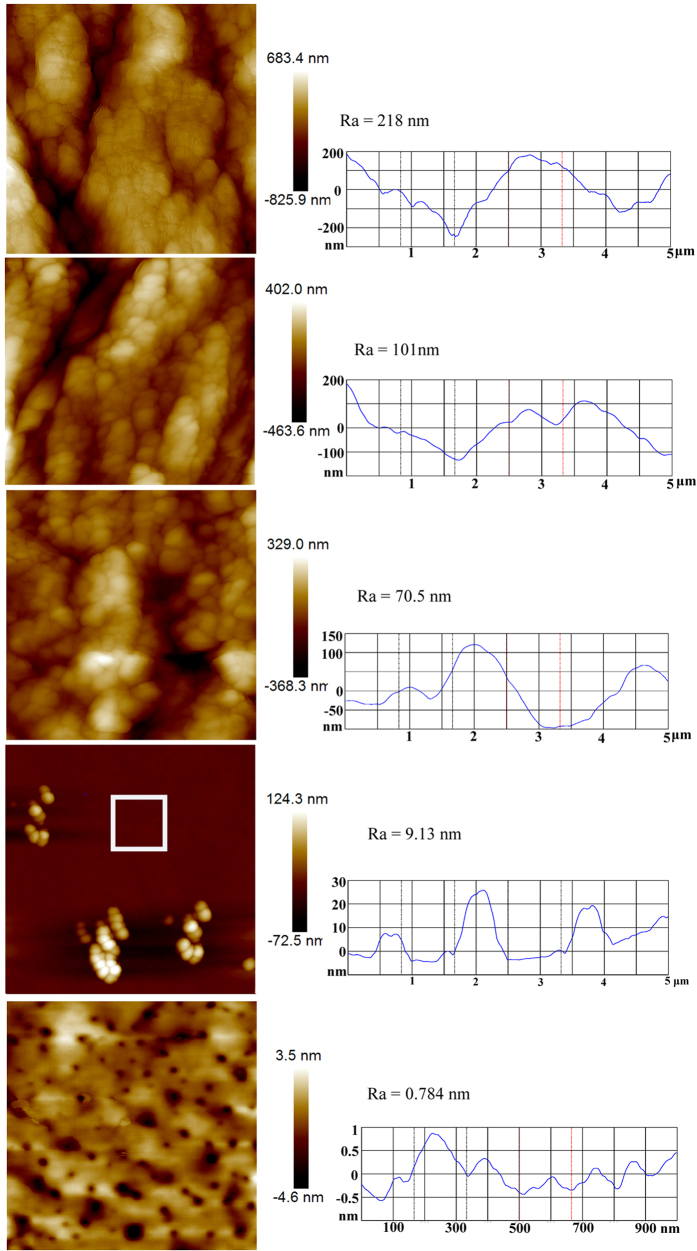
AFM height images and roughness values of the surface of different group samples. Pane (a) is the acid-etched enamel surface. Pane (b) is the FC-70-adsorbed surface. Pane (c) is the polyfluoroalkyl-silanized surface. Pane (d) is the SLIPS. Pane (e) is the magnification of “□” part of pane (d). Panes (a–d) are the size of 5 μm × 5 μm. Pane (e) is the size of 1 μm × 1 μm. The balls in pane (d) and the holes in pane (e) may come from the compress air during the process of preparing the samples for AFM.

In this study, AFM was used to investigate the morphologies and surface roughness values of the enamel surfaces (Fig. 1). The acid-etched enamel surface showed a very rough morphology of ridges and valleys with micro/nanoparticles. The average root mean square roughness (Ra) was about 218 nm (5 μm × 5 μm sizes) (Fig. 1a). For the samples of lubricant FC-70 directly adsorbed to the acid-etched enamel surface (FC-70-adsorbed surface), the surface morphology was similar to the acid-etched enamel surface; however, average roughness values decreased to Ra = 101 nm, which may be caused by FC-70 trapped in the valley (Fig. 1b). For the samples of (perfluorooctylethyl)trichlorosilane-grafted acid-etched enamel surface (polyfluoroalkyl-silanized surface), the morphology of the acid-etched enamel structure could still be observed, but the average roughness values decreased greatly to Ra = 70.5 nm (Fig. 1c). For the samples of the lubricant FC-70-infused polyfluoroalkyl-silanized surface (SLIPS), the morphology was very smooth with a very low roughness value of Ra = 9.13 nm, and the acid-etched enamel structure could not be found (Fig. 1d). In the image of size of 1 μm × 1 μm, the roughness value was 0.784 nm (Fig. 1e). Therefore, roughness analysis confirmed that the lubricating fluid overcoated the surface topographies of the porous enamel surface, forming a nearly molecularly smooth surface.

**Figure 2:**
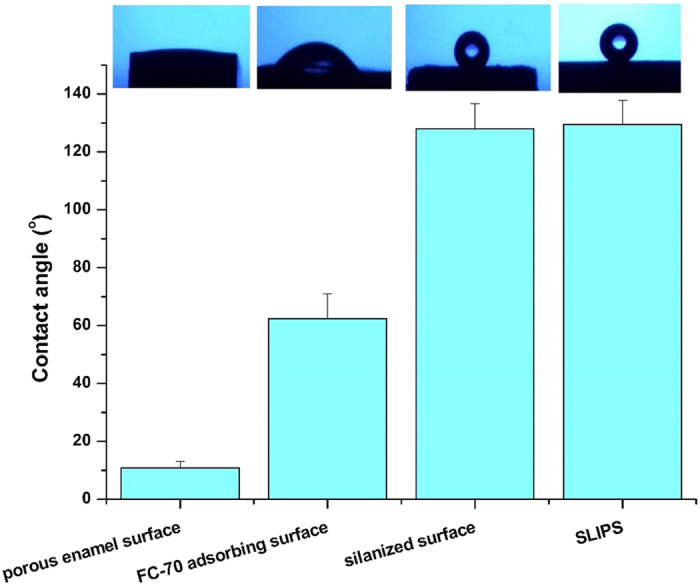
Contact angle of different groups.

The water contact angles measured on acid-etched enamel surface, FC-70 directly adsorbed acid-etched enamel surface, (perfluorooctylethyl) trichlorosilane grafted acid-etched enamel surface (hydrophobic polyfluoroallkyl silanized surface) and lubricant FC-70 infused polyfluoroalkyl-silanized surface (SLIPS) were shown in Fig. 2. The results showed the differences among different groups were statistically significant [F (3, 20) = 377.15; p < 0.05], which indicated that the silanized surface and SLIPS became hydrophobic, but the FC-70 directly adsorbed surface and acid-etched enamel surface remained hydrophilic. Statistically, the contact angles between different groups were significantly different (p < 0.05), except those between the silanized surface and SLIPS groups (p > 0.05).

**Figure 3:**
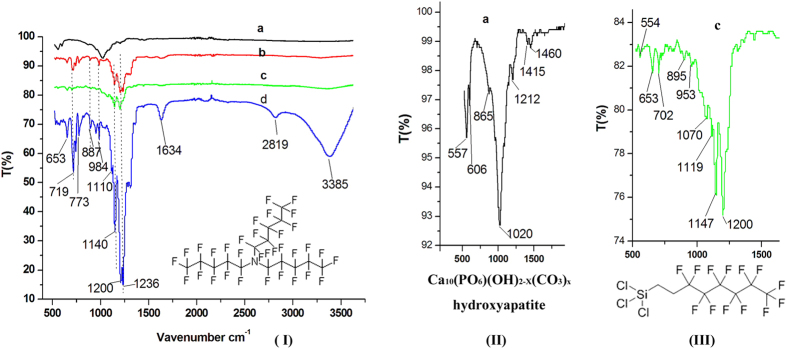
(**I**) DR-FTIR spectra of acid-etched enamel surface (hydrophilic micro/nanoporous enamel surface) (a), acid-etched enamel surface trapped with FC-70 (control) (b), acid-etched enamel surface grafted with (perfluorooctylethyl)trichlorosilane (hydrophobic polyfluoroalkyl-silanized surface) (c), and lubricant FC-70 infused into polyfluoroalkyl-silanized surface (SLIPS) (d). (**II**) Magnification of FTIR spectrum of “(I) (a)” between 500 and 2000 cm^−1^. (**III**) Magnification of FTIR spectrum of “(I) (c)” between 500 and 2000 cm^−1^.

In terms of composition and structure, the FTIR spectra confirmed that FC-70 covered the enamel surface (Fig. 3). In the FTIR spectrum of the acid-etched enamel surface, the peaks at 1020, 557 and 606 cm^−1^ were distinctive of the -PO_4_ group, whereas the peaks at 1415, 1460, 1212 and 865 cm^−1^ were attributed to -CO_3_. These findings suggested that the acid-etched enamel surface was composed of carbonate containing hydroxyapatite. In the spectrum of the acid-etched enamel surface grafted with (perfluorooctylethyl)trichlorosilane, peaks at 1200, 1147, 1119, 1070, 953, 899, 702 and 652 cm^−1^ were attributed to –CF_2_ and –CF_3_ groups on different sites or Si-O and Si-O-Si groups for overlapping. However, the peak at 554 cm^−1^ was attributed to the Si-O group^19^,^20^,^21^. Based on the FTIR findings and contact angle changes, we confirmed that (perfluorooctylethyl)trichlorosilane was grafted to the enamel surface. In the sample of lubricant FC-70 infused into the polyfluoroalkyl-silanized surface (SLIPS), peaks at 1236, 1200, 1140, 1110, 984, 877, 773, 719 and 653 cm^−1^ were attributed to the –CF_2_ and CF_3_ groups on different sites, whereas peaks at 3385 and 1634 cm^−1^ were attributed to C-N binding^19^,^20^,^21^; the magnitude of peaks caused by C-N binding was much stronger than the magnitude of other peaks. These results suggested that FC-70 existed on the sample surface. By contrast, in the sample of the acid-etched enamel surface directly adsorbing FC-70, the peaks attributed to the –CF_2_ and CF_3_ groups were found, but their strength was much smaller than the peaks from the slippery liquid-infused enamel surface. Thus, the content of FC-70 on the enamel surface was much smaller than that of SLIPS, and FC-70 may be trapped into the pores of the porous enamel surface.

### SLIPS inhibits mucin absorption, *S. mutans* adhesion and biofilm formation *in vitro*

**Figure 4:**
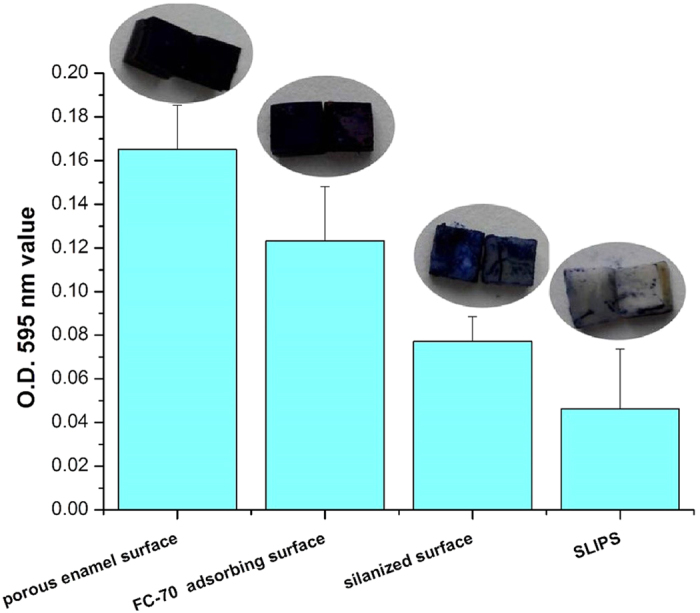
Mucin absorption in different surfaces. The images above the bars of each group are the typical surface color of each group samples stained by Alcian blue.

The amount of mucin absorbed by the different enamel surfaces is shown in Fig. 4. The samples in SLIPS group were stained lightest by Alcian blue solution. And, a statistically significant difference was detected among all the groups (p < 0.05). Mucin absorbed by the slippery liquid-infused enamel surface was considerably less than that by the other groups. The sequence of the amount of absorbed mucin was as follows: slippery liquid-infused enamel surface < polyfluoroalkyl-silanized surface < FC-70 adsorbed surface < acid-etched porous enamel surface.

**Figure 5:**
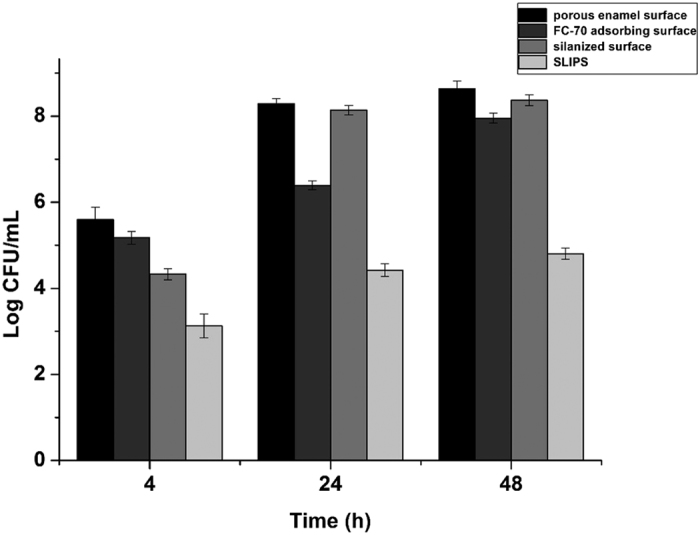
Bacterial counts (log CFU/mL) after 4, 24 and 48 h in different groups.

The quantity of bacteria adhered and grown on each sample of different groups was evaluated by CFU counts of the biofilm, and the results are shown in Fig. 5. The log CFU value increased as the culture time increased from 4 h to 24 and 48 h in every group. The one-way ANOVA results showed that the differences among different groups at the same culture stage were statistically significant (p < 0.05), which indicated that the average bacterial counts of different groups were not all equal. Furthermore, Student-Neuman-Keuls post-hoc test showed that the differences between any two groups at the same culture stage were statistically significant (p < 0.05), which meant that the average bacterial counts of between any two groups at the same culture stage were not equal. The values of the SLIPS group were considerably lower than those of the control groups. On the other hand, the one-way ANOVA results showed that the differences among different culture times in the same group were statistically significant (p < 0.05), which indicated that the average bacterial counts of the same group at the different culture stage were not all equal. Furthermore, Student-Neuman-Keuls post-hoc test showed that the differences between any two culture stages in the same group were statistically significant (p < 0.05), which meant that all of the average bacterial counts of the same group at between any two culture stage were not equal.

**Figure 6:**
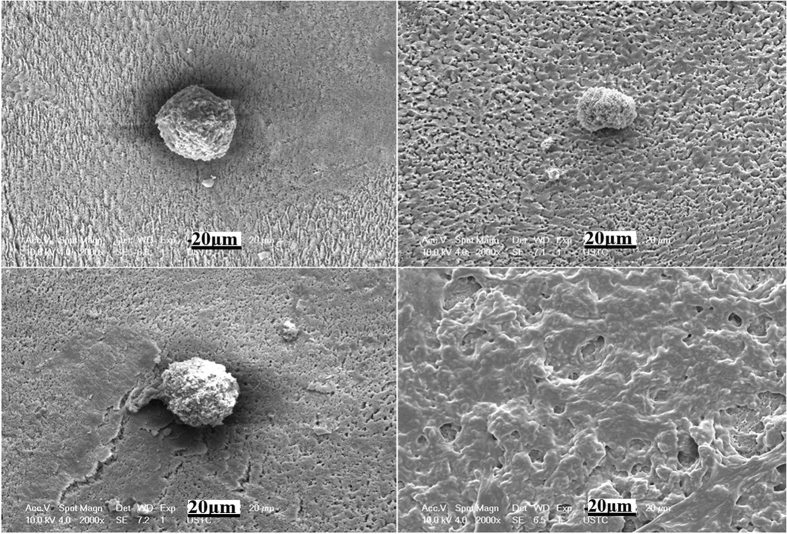
SEM images of biofilm morphology on the sample surfaces of different groups after 4 h. Pane (a) is the acid-etched porous enamel surface, pane (b) is the FC-70-adsorbed surface, pane (c) is the polyfluoroalkyl-silanized surface, and pane (d) is the SLIPS.

**Figure 7:**
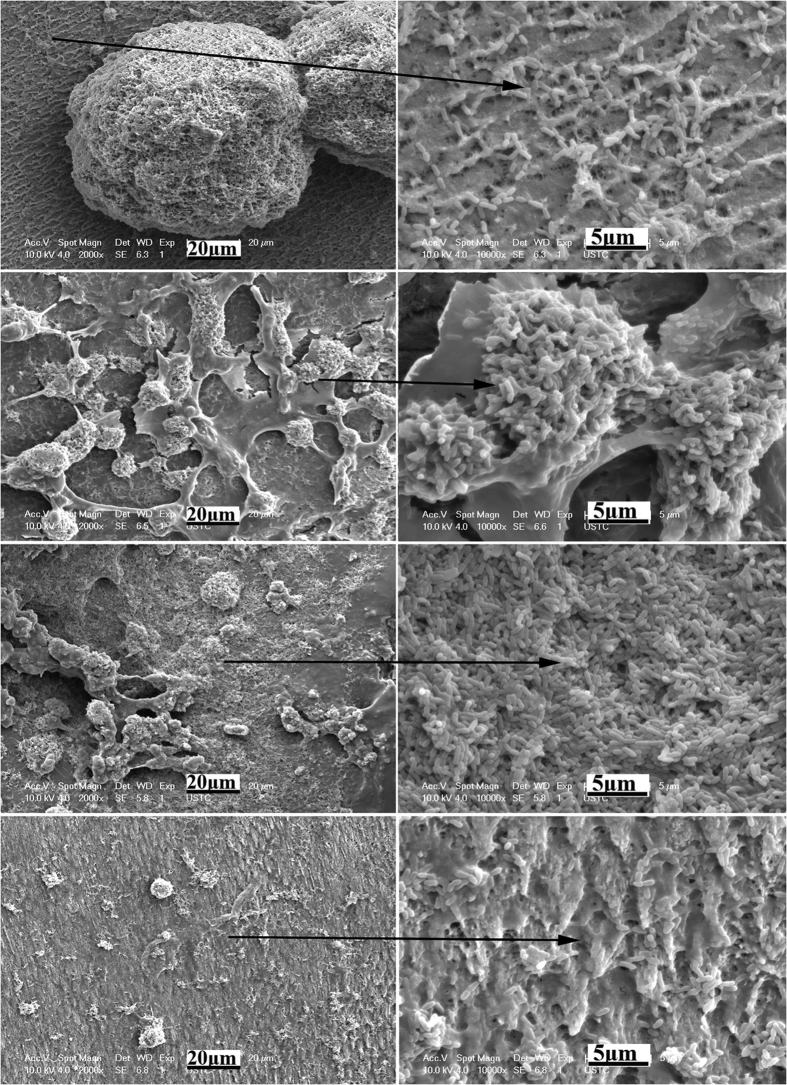
SEM images of biofilm morphology on the sample surfaces of different groups after 24 h. Pane (a) is the acid-etched porous enamel surface, pane (b) is the FC-70-adsorbed surface, pane (c) is the polyfluoroalkyl-silanized surface, and pane (d) is the SLIPS. Panes (a_1_–d_1_) are the magnification of the parts of panes (a–d) respectively.

**Figure 8:**
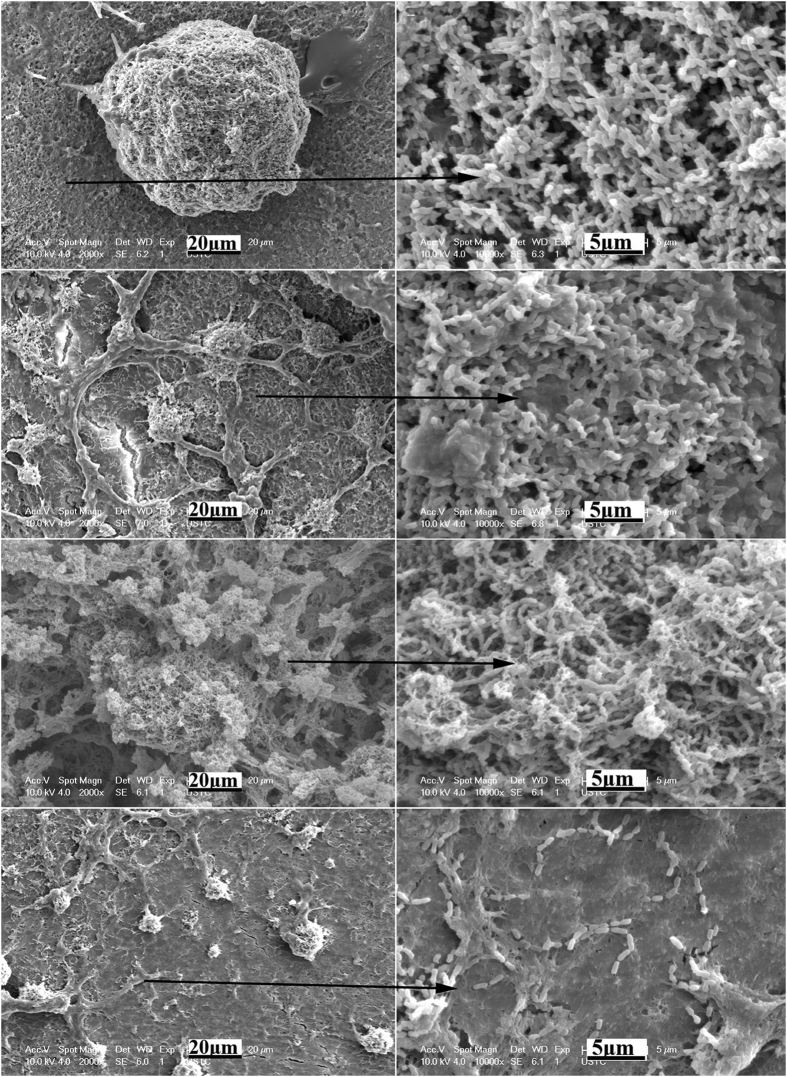
SEM images of biofilm morphology on the sample surfaces of different groups after 24 h. Pane (a) is the acid-etched porous enamel surface, pane (b) is the FC-70-adsorbed surface, pane (c) is the polyfluoroalkyl-silanized surface, and pane (d) is the SLIPS. Panes (a_1_–d_1_) are the magnification of the parts of panes (a–d) respectively.

To qualitatively evaluate the bacteria adhered and grown on each samples of different groups, the biofilm morphology was analyzed by SEM, and the results are shown in Figs 6, 7, 8. For the 4 h samples, bacteria could rarely be found on the surface of the SLIPS group (Fig. 6d), whereas bacteria were distributed in globular morphology on all the control groups of the acid-etched porous enamel surface, FC-70-adsorbed surface, and polyfluoroalkyl-silanized surface (Fig. 6a–c). For the acid-etched porous enamel surface, bacteria were sparsely distributed in some areas. However, enamel surfaces were still visible in all the control groups.

After 24 h of bacterial growth, only sparse and isolated bacteria were observed on the SLIPS (Fig. 7d,d1). However, for all the control groups, the surfaces were covered by a monolayer or multilayer of crosslinked biofilm (Fig. 7a–c). The acid-etched porous enamel surface and FC-70-adsorbed surface grew and accumulated more bacteria than the polyfluoroalkyl-silanized surface.

After 48 h, bacteria were still sparsely isolated and distributed on the SLIPS with minimal growth compared with that at 24 h (Fig. 8d). Meanwhile, the surfaces of all control groups were covered by thick, robust, and uniform biofilms, which comprised a mixture of bacteria arranged in a multilayered matrix structure (Fig. 8a–c). The hydrophobic polyfluoroalkyl-silanized surface still accumulated more bacteria than the acid-etched enamel surface.

### SLIPS inhibits dental biofilm development *in vivo*

**Figure 9:**
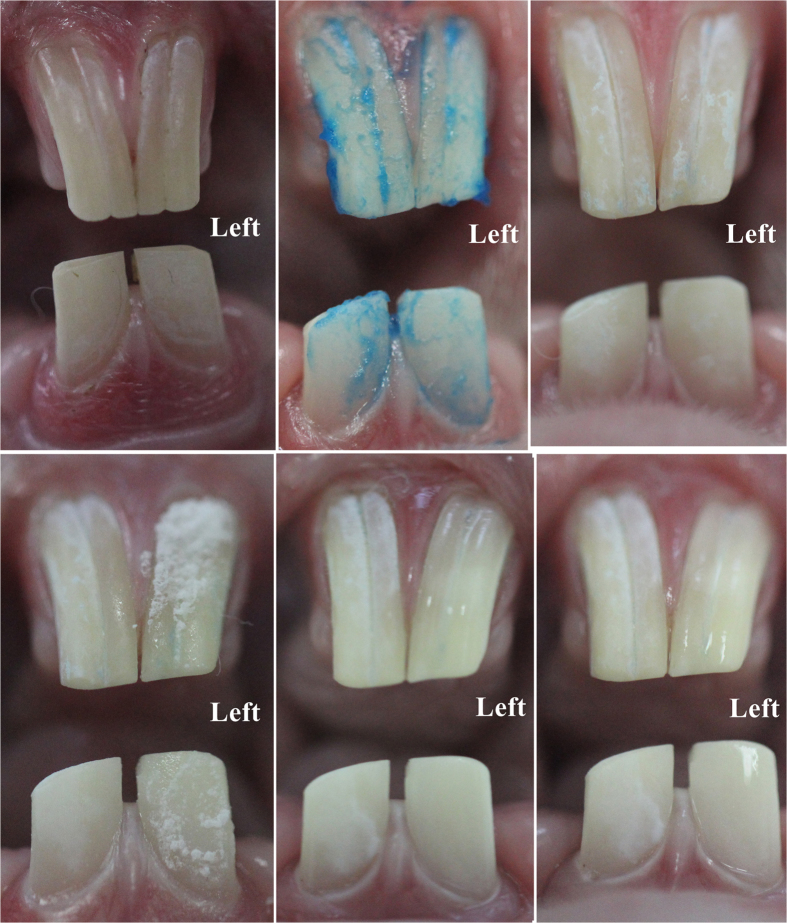
Process of fabrication of SLIPS on rabbit incisors. Pane (a) shows the absence of fuchsin stain on the rabbit incisor surface before SLIPS fabrication, thereby suggesting that dental plaque was removed by a mechanical toothbrush. Pane (b) shows the 20% phosphoric acid gel etched the enamel surface, and pane (c) shows the acid-etched enamel surface with chalky white appearance after rinsing with water. Pane (d) shows the acid-etched enamel surface applied with polyfluoroalkyl silane, and its appearance after rinsing with ethanol (**e**). Pane (f) shows FC-70 added to the silanized surface to form the SLIPS.

**Figure 10:**
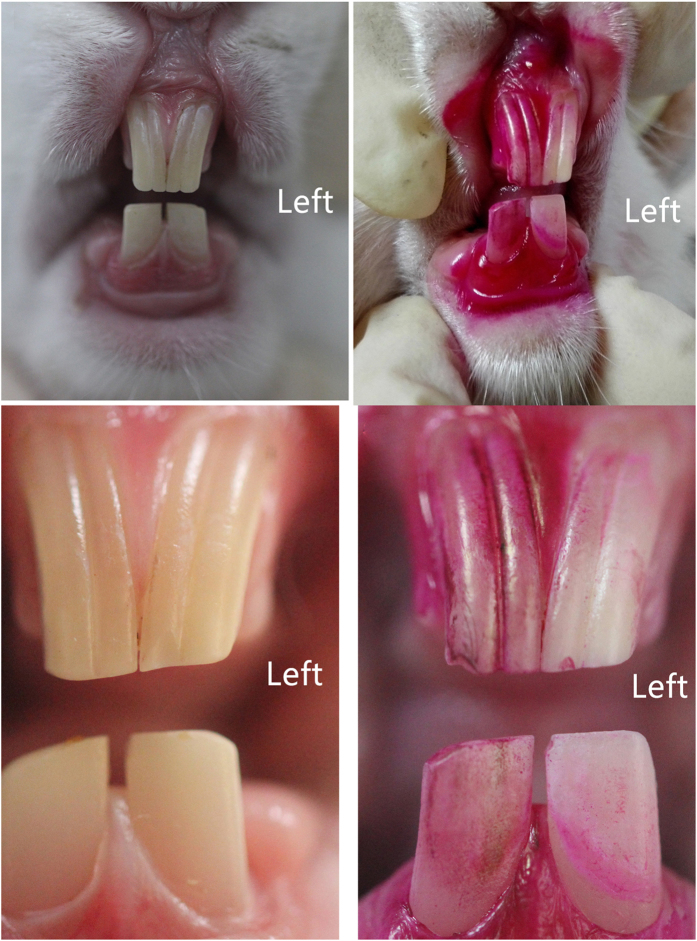
Surface appearance after 10% fuchsin stain before (**a**,**c**) and after SLIPS fabrication for a 48 h high sucrose diet (**b**,**d**). No stain before SLIPS fabrication (**a**,**c**). After a 48 h high sucrose diet, a small stain was observed on the SLIPS (left incisors), whereas many stains were found on the acid-etched incisor surface (right incisors).

Figure 9 shows the process of SLIPS fabrication on left incisor surfaces of rabbits. Before fabricating SLIPS, incisors were cleaned with no stains of plaque indicator (Figs 9a and 10a), which suggested that all the dental plaque on the incisor surfaces had been removed. To construct a porous enamel surface for SLIPS fabrication, 20% phosphoric acid gel was applied to the labial surfaces of the incisors (Fig. 9b), and the chalk white appearance suggested that a rough surface formed (Fig. 9c). Heptadecafluoro-1,1,2,2-tetra-hydrodecyltrichlorosilane was added to the acid-etched porous surface and then grafted to the enamel surface by hydrolysis (Fig. 9d). Ethanol was used to remove the surplus polyfluoroalkyl silane (Fig. 9e). The functional polyfluoroalkyl-silanized surface matched the chemical nature of FC-70, resulting in infiltration to form the SLIPS (Fig. 9f).

Figure 10 shows the effect of the SLIPS on inhibiting dental plaque development. The surfaces of incisors with SLIPS developed significantly less dental plaque than in the acid-etched enamel surface after a 48 h high sucrose diet.

## Discussion

The SLIPS inspired by the leaves of a pitcher plant, exhibits remarkable properties such as liquid repellency, smoothness, and self-healing and antibiofouling activities^14^,^15^. A stable SLIPS, which consists of a film of lubricating liquid locked in place by a micro/nanoporous substrate, is designed based on three important criteria: (i) the solid should preferably be roughened to increase the surface area for adhesion of the lubricating fluid and its immobilization; (ii) the chemical affinity between the lubricating fluid and solid should be higher than that between the ambient fluid and solid; and (iii) the lubricating fluid and ambient fluid must be largely immiscible^18^. To satisfy the first requirement in constructing the slippery liquid-infused enamel surface, a micro/nanotextured rough surface with a large surface area was obtained by acid etching, which is a commonly used technique in clinical dentistry. The micro/nanotextured rough surface facilitated wicking the lubricating liquid into the enamel surface. To satisfy the second criterion, the acid-etched hydrophilic rough surface was functionalized using hydrophobic low-surface energy polyfluoroalkyl silane to match the chemical nature of the infiltrated lubricant chosen to be immiscible with the ambient fluid. In the experiment, the surface tension of (perfluorooctylethyl)trichlorosilane is about 17.2 N/cm, and that of lubricant FC-70 is about 13.8 N/cm^18^. The trichlorosilane residual groups of (perfluorooctylethyl) trichlorosilane easily react with the hydroxyl groups of the enamel surface, so that the polyfluoroalkyl tail is grafted on the enamel surface to make the enamel surface hydrophobic. Thus, the polyfluoroalkyl-silanized enamel surface displayed more affinity to the lubricant than the ambient hydrophilic fluid. For the third criterion, the lubricant is hydrophobic but the ambient fluid is hydrophilic, so they are immiscible. The results of FTIR, AFM and contact angle all confirmed that the super hydrophobic slippery liquid-infused enamel surface was created. During the process of constructing SLIPS, polyfluoroalkyl silanization of the acid-etched rough enamel surface is very important. Without polyfluoroalkyl silane, lubricant FC-70 could not be evenly infused into the porous enamel surface because of the immiscibility of the hydrophilic enamel surface and the hydrophobic FC-70. Although FTIR could detect FC-70 on the surface of FC-70-adsorbed samples, some FC-70 may only be trapped into the pores or the valley of the rough enamel surface. AFM images, contact angles, and the results of antibiofouling test *in vitro* suggested that SLIPS was not formed without polyfluoroalkyl silane pre-treatment of the rough enamel surface.

**Figure 11:**
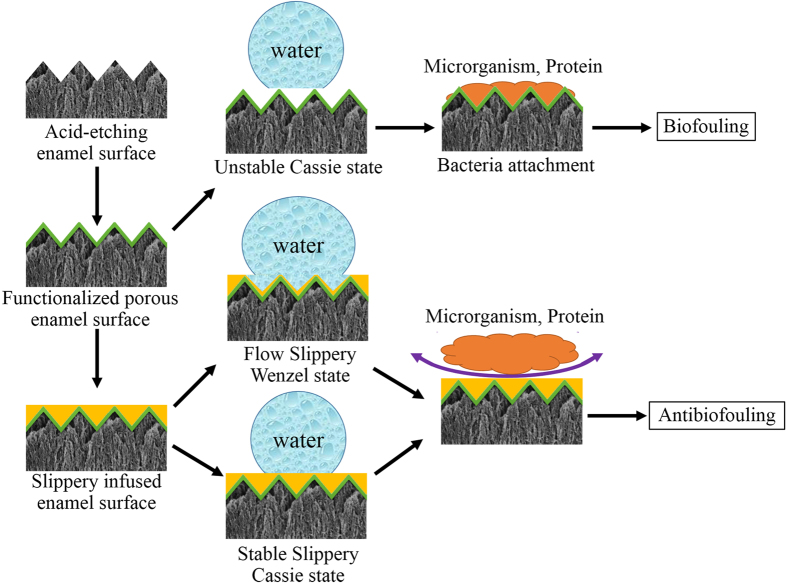
Scheme of constructing the slippery infused enamel surface and its mechanism of antibiofouling property.

The strategy of constructing a bacteria-resistant dental surface without using bactericidal compounds has opened a new way to prevent dental biofilm formation. An antibiofouling dental surface was constructed by grafting a derivative of pyrophosphate (PPi)/phosphate (Pi)-PEG to the dental surface^22^,^23^. The PPi/Pi groups bind to the enamel surface and occupy the saliva protein-binding site of positively charged calcium to inhibit saliva protein adsorption to the enamel surface. Moreover, the polymer brush of PEG creates a neutral hydrophilic layer, which effectively reduces the hydrophobic interactions of salivary protein and bacteria with the enamel surface. On the other hand, hydrophobic varnishes applied to teeth has been proved to prevent bacteria adhesion^24^. However, the traditional antibiofouling surfaces are fundamentally in solid forms by surface chemistry or surface structuring. Permanent interactions between the solid surfaces and biological adhesives are eventually established, which can lead to stable attachment and biofouling. Additionally, any defects in surface chemistry can serve as nucleation sites for bacterial attachment. The long-term effect of PEG for antibiofouling is limited, and SLIPS is highly effective^18^. SLIPS processes dynamic features down to the nanometer scale. A stable, immobilized, and smooth liquid surface locked in place by a specially designed porous solid provides a nonadhesive slippery interface that is independent of any specific chemical or physical features. On SLIPS, bacteria are presented with a smooth liquid “surface”, so they may be unable to anchor to the mobile interface via pili and other cellular mechanisms as would be possible on a solid surface, thereby significantly disrupting biological adhesion. Additionally, SLIPS is a nontoxic and nonbiocidal antibiofilm synthetic surface^18^. In the present study, the enamel surface with SLIPS showed superior antibiofouling property in inhibiting dental biofilm formation *in vitro* and *in vivo*. The acid-etched enamel surface could be easily absorbed by the salivary protein of mucin and adhered by *S. mutans*. Biofilms grew and matured on the enamel surfaces with increasing culture time. After the acid-etched surface was pretreated by polyfluoroalkyl salinization, mucin adsorption and *S. mutans* adhesion at the initial time were inhibited (Figs 4, 5 and 6). The hydrophobic surface property may contribute to this phenomena. However, as above discussion, this anti-biofouling surface was in solid form, which cannot inhibit the permanent interactions between the solid surfaces and the biological adhesives, and eventually lead to stable attachment and biofouling. On the other hand, the polyfluoroallkylized rough enamel surface may be hydrophobic because water droplets are in the Cassie state, of which liquid droplets sit on top of rough solid textures and air is trapped underneath. Sustaining a droplet in the Cassie state is difficult, as the air layer underneath the droplets can be disrupted, such as in liquids with low surface tension or liquids with impurities^25^,^26^. Thus, structured hydrophobic surfaces in the Cassie (trapped air) state are prone to irreversible wetting (Wenzel transition), especially with the production of bacterial surfactants, seriously limiting their lifetime in submerged environments^25^. Therefore, we observed that *S. mutans* biofilm grew and matured on the polyfluoroalkylized enamel surface after 48 h of culture (Figs 5, 7 and 8). Fornell AC *et al.* also reported that hydrophobic polymer coating had less clinically beneficial effect in a low-caries group of adolescents^24^. For the samples of FC-70 directly adsorbed surfaces, slippery lubricant FC-70 was only trapped in some pores or valley of the acid-etched enamel surface because of the immiscibility of the hydrophilic enamel surface and the hydrophobic FC-70. Thus, the poor antibiofouling property of FC-70 directly adsorbed samples is not difficult to understand (Figs 4, 5, 7 and 8). In the SLIPS samples, we observed that a few bacteria adhered but the bacteria grew very slowly, and the biofilm was not mature after 48 h (Figs 5, 8 and 10). The CFU count of SLIPS after 48 h was even smaller than that of the acid-etched enamel surface after 4 h (p < 0.05) (Fig. 4). In the polyfluoroalkyl-salinized enamel surface, liquid droplets were mobile in the Cassie state and pinned in the Wenzel state (the drops impregnated the solid textures). In the SLIPS condition, Wenzel state droplets still exhibited high droplet mobility on rough microtextured surfaces. A chemically homogeneous layer of lubricant infused into the nanotextures of a hierarchically structured surface. The sharp edges were smoothened by the liquid lubricant, yielding a conformally lubricated rough surface. The pinning effect on such surfaces could be greatly reduced^25^. Herein, we stress on the difference between the hydrophobic solid surface and SLIPS constructed on a hydrophobic surface. The structure and antibiofouling mechanism of the SLIPS are shown in Fig. 11.

Saliva protein adsorption changes the physicochemical property of the tooth surface, adds specific receptors for microbial adhesion, and influences bacterial adhesion. Mucin is one of the major components in human saliva to act as a receptor for accumulation and biofilm formation; it also plays an important role in agglutination/aggregation of a number of microorganisms^27^,^28^. Oral streptococci pioneer early dental plaque formation and play an important role in the development of oral biofilms. *S. mutans* is considered the most cariogenic of all oral streptococci because of its acidogenic property, acid tolerance, and ability to synthesize extracellular glucans^29^. Furthermore, it plays an important role in the adhesion of other bacteria. Therefore, we selected mucin and *S. mutans* as the model to evaluate the antibiofouling property of the enamel surface with the SLIPS *in vitro*. From our experimental observations, mucin and *S. mutans* were scarcely absorbed by the slippery liquid-infused enamel surface. The experiments clearly showed poor biofilm attachment to the SLIPS compared with the other surfaces *in vitro*; however, the effects *in vivo* must be determined. In our experiments, the SLIPS was available and effective for at least 48 h in rabbits. However, the observed effectiveness during 48 h still poses clinical significance for patients who are unable to perform strict oral hygiene because of trauma or surgery. This study is the first to explore the possibility of the SLIPS applied to tooth surfaces.

Further attention should be paid to the long-term effectiveness of the SLIPS on teeth. According to the present study *in vitro* and *in vivo*, it is clear that bacteria are present, albeit in smaller numbers on the SLIPS surfaces, and these could presumably go on to form more substantial biofilms if left for a longer period. However, after 48 h, bacteria were still sparsely isolated and distributed on the SLIPS with minimal growth or in globular morphology on SLIPS *in vitro* (Fig. 8d). It may suggest that SLIPS effectively disrupt biological adhesion. As above discussion, SLIPS processes dynamic nanometer scale smooth liquid surface so that bacteria may be unable to anchor to the mobile interface via pili and other cellular mechanisms as would be possible on a solid surface. However, the environment of oral cavity is much more complex, in which around 500 species of oral microbial communities present a structurally and dynamically complex ecosystem where polymicrobial biofilms are the norm^10^. Therefore, it is much more difficult to prevent biofilm formation on tooth surface *in vivo* than *in vitro*. Though in the rabbit model, dental plaque was greatly reduced on the SLIPS enamel surface after 48 h, there was still some dental plaque on the SLIPS enamel surface. On the other hand, it is very necessary to pay attention to the stable property of the SLIPS on enamel surface. The chewing and the tooth brush may disrupt the integrity of the SLIPS. So, there are still a lot of researches need to be done before application.

## Conclusion

The slippery liquid-infused enamel surface was constructed by acid etching and then functionalized by hydrophobic low-surface energy polyfluoroalkyl silane upon wicking the lubricating liquid into the surface. The novel slippery liquid-infused enamel surface could inhibit saliva protein and bacterial adhesion *in vitro* and *in vivo*, and may potentially provide a novel way to prevent dental biofilm formation.

## Materials and Method

### Materials

Heptadecafluoro-1,1,2,2-tetra-hydrodecyltrichlorosilane (96%) and perfluorotriamylamine (FC-70) (85%) were purchased from Aladdin Industrial Company (Shanghai, China). Brain heart infusion (BHI) was obtained from Oxoid (Basingstoke, UK). All other chemicals were purchased from Sigma–Aldrich (St. Louis, MO, USA) unless otherwise noted.

### Specimen preparation

Fresh bovine incisors were obtained from a slaughterhouse. The soft tissue attached to the teeth was removed with a scalpel. The teeth were treated with 3% sodium hypochlorite to remove bacteria and then rinsed with 1% (v/v) phosphate-buffered saline (PBS, pH 7.2). Tooth slices of 2 mm thickness were cut by a water-cooled diamond saw (IsoMet low-speed saw, Buehler, Lake Bluff, IL, USA). The enamel blocks of 4 × 4 × 2 mm^3^ were prepared. A total of 100 enamel slices without cracks were selected for use in this study. The slices were polished with 600-, 1200-, 2400-, and 4000-grit silicon carbide papers and then ultrasonically cleaned with acetone, ethanol, and deionized water. They were stored in distilled water at 4 °C until further use.

### Slippery liquid-infused enamel surface preparation

Enamel slices were etched by 37% phosphoric acid for 1 min, washed with copious deionized water, and dried with nitrogen to obtain a micro/nanoporous enamel surface. Acid-etched porous enamel slices were immersed in 2 mL of heptadecafluoro-1,1,2,2-tetra-hydrodecyltrichlorosilane solution for 30 min in a closed container, washed with ethanol three times for 5 min each, and dried with nitrogen to obtain the polyfluoroalkyl-silanized enamel surface. The silanized enamel slices were immersed in 2 mL of FC-70 for 30 min in a closed container. The samples were tilted, and a stream of nitrogen was used to assist the removal of excess lubricant to obtain the slippery liquid-infused enamel surface.

To evaluate the character of the slippery liquid-infused enamel surface, three control groups were prepared, namely, control groups 1, 2 and 3. Control group 1 comprised an acid-etched porous enamel surface (substrate: porous enamel surface). Control group 2 was polyfluoroalkyl-silanized acid-etched porous enamel surface (polyfluoroalkyl-silanized surface). Control group 3 was an acid-etched porous enamel surface without polyfluoroalkyl silanization. The surface was directly immersed into FC-70 for 30 min, and excess lubricant was removed with a stream of nitrogen (FC-70 adsorbed surface).

### Surface characterization

The morphology and roughness of the samples were characterized by atomic force microscopy (AFM) analysis using a tapping model with an etched silicon probe (Dimension Edge, Bruker, CA, USA). The chemical properties of the surface were evaluated by diffuse-reflectance Fourier transform infrared spectroscopy (DR-FTIR) (Nicolet 8700, Thermo Scientific Instrument Co., Friars Drive Hudson, NH, USA). Surface wettability was expressed with contact angle and evaluated on a JGW-360A1 (City-Hui Test Machine Co, Chengde, China) equipped with a pendant drop module. Contact angles were subtended by water drops on the flat specimen surfaces, and these droplets were captured using a video and transferred to a computer for angle measurement. For each sample, three different areas were selected to measure the contact angles. For all above examination, two samples per groups were analyzed and the experiment were repeated for three times.

### *In vitro* inhibition of salivary protein of mucin adhesion to the slippery liquid-infused enamel surface

After immersion in 10 mg/mL mucin from bovine submaxillary glands for 90 min at 37 °C, specimens (experiment group: slippery liquid-infused enamel surface, and three control groups as above, n = 6) were obtained, rinsed with PBS, and stained with Alcian blue solution for 90 min at about 37 °C. Immediately after rinsing with 1% PBS for three times, the enamel blocks were each placed into 1 mL of PBS. After centrifugation at 3500 rpm for 10 min, the optical density of the supernatant (20 μL) was measured spectrophotometrically at 595 nm (Bio Tek Instruments Co., VT, USA)^27^.

### *In vitro* inhibition of *Streptococcus mutans* adhesion and biofilm formation on the slippery liquid-infused enamel surface

*S. mutans* American Type Culture Collection 35668 was cultured on BHI at 37 °C for 2 days anaerobically. A single colony was selected from a plate to prepare 24 h broth cultures in BHI supplemented with 5% sucrose at 37 °C under anaerobic conditions (80% N_2_, 10% CO_2_, and 10% H_2_). Subsequently, bacterial cell pellets were harvested and resuspended in BHI to a cell density of McFarland 2 (10^6^ cells/mL). 100 μL of bacterial culture was mixed and inoculated on each enamel block sitting in a well of a 24-well plate with 900 μL of BHI. Enamel blocks were incubated in 1 mL of mixed suspension aerobically for 90 min (adhesion phase) at 37 °C and then gently rinsed twice with PBS to remove non-adherent cells^30^. Subsequently, enamel blocks were aerobically incubated in BHI at 37 °C (biofilm phase) and taken out for analysis after 4, 24 and 48 h.

Each group contained 8 samples. Two samples were used for the following scanning electron microscopy (SEM) observation, and the other six samples were used for the total bacterial colony forming unit (CFU) counting.

SEM was used to examine the topographical features of *S. mutans* adhesion and biofilm. In preparation for SEM, enamel blocks with biofilm were placed in 2.5% (v/v) glutaraldehyde solution overnight at 4 °C. The blocks were washed in distilled water and dehydrated in a series of ethanol solutions at increasing concentrations (50% for 10 min, 70% for 10 min, 95% for 10 min and 100% for 20 min). The samples were then dried in a critical-point evaporator before sputter coating with gold for SEM analysis (FE-SEM, Sirion 200, FEI Co, Hillsboro, OR, USA).

To determine the total bacterial colony forming unit (CFU) after 4, 24 and 48 h, each specimen was removed aseptically and washed three times with PBS to remove loosely adherent cells. The specimens were then transferred to a tube containing a liquid BHI culture medium. The remaining adherent microorganisms were removed from the enamel blocks by sonication (Hefei Kinnick Machinery Manufacturing Co., Hefei, China) for 20 min. 100 μL resultant suspension was diluted by 900 μL 1% PBS. The serial 10-fold dilutions of the resultant suspension were plated in duplicate onto BHI agar. The plates were incubated anaerobically for 72 h. The CFU counting results were checked by a technician who was blinded to the experiment design^31^.

### Inhibition of dental biofilm development on SLIPS *in vivo*

Six healthy New Zealand white rabbits aged 6–9 months and weighing 2.2–2.6 kg were used in this experimental study (The Center of Experimental Animals, Anhui Medical University, Hefei, China). All of the experimental procedures involving animals were performed under a protocol that was reviewed and approved by the Ethics Committee of Anhui Medical University (Permit number: 20150251). All of the animal work was conducted in accordance with national and international guidelines to minimize animal suffering. All procedures were carried out under general anesthesia, and animals were injected with 3% sodium pentobarbital (1 mL/kg) into the lateral ear vein. In this experiment, the left incisors were administered on the SLIPS and the right incisors were used as control. First, both the left and right incisors were cleaned with a toothbrush until the labial surfaces of incisors were no longer dyed by plaque indicator (2% fuchsin solution). Phosphoric acid gel (Gluma, Heraeus Kulzer Dental Co., Hanau, Germany) was applied to the labial surfaces of the left incisors for 60 s. The etchant was rinsed off, and the teeth were dried thoroughly. A thin layer of heptadecafluoro-1,1,2,2-tetra-hydrodecyltrichlorosilane solution was coated to the frosty white enamel surfaces in the form of droplets. After 60 s, 10 mL of absolute ethyl alcohol was used to wash the enamel surfaces of the left incisors. Finally, FC-70 was coated to the surfaces in the form of droplets for 60 s to obtain SLIPS on the enamel surface. The animals were fed with a high sucrose diet for the following 48 h^22^,^32^. Dental plaque indicator was used again to indicate the plaque in both the SLIPS group and control group.

### Statistical analysis

Statistical tests were performed using the SPSS 17.0 program. Data were assessed for a normal distribution using the Shapiro–Wilk test for normality (p > 0.05), in which CFU values were converted to log10. One-way ANOVA was used to compare the mucin absorption and CFU values of the four experimental groups. Results showing significant overall changes were subjected to Student-Newman-Keuls post- hoc test. The cut-off level for significance was taken as 5% for all of the analyses.

## Additional Information

**How to cite this article**: Yin, J. L. *et al.* Self-cleaning and antibiofouling enamel surface by slippery liquid-infused technique. *Sci. Rep.* **6**, 25924; doi: 10.1038/srep25924 (2016).
